# Genetic and environmental risk factors for rheumatoid arthritis in a UK African ancestry population: the GENRA case–control study

**DOI:** 10.1093/rheumatology/kex048

**Published:** 2017-04-12

**Authors:** Matthew Traylor, Charles Curtis, Hamel Patel, Gerome Breen, Sang Hyuck Lee, Xiaohui Xu, Stephen Newhouse, Richard Dobson, Sophia Steer, Andrew P. Cope, Hugh S. Markus, Cathryn M. Lewis, Ian C. Scott

**Affiliations:** 1 Department of Medical and Molecular Genetics, King’s College London; 2 SGDP Centre, Institute of Psychiatry, Psychology & Neuroscience, King’s College London; 3 Department of Rheumatology, King’s College Hospital; 4 Academic Department of Rheumatology, Centre for Molecular and Cellular Biology of Inflammation, King’s College London, London; 5 Department of Clinical Neurosciences, Neurology Unit, University of Cambridge, Cambridge, UK; a Present address: Department of Rheumatology, Haywood Hospital, Burslem, UK.; b Present address: Research Institute for Primary Care & Health Sciences, Keele University, Staffordshire, UK.

**Keywords:** arthritis, rheumatoid, African continental ancestry group, genetic susceptibility, smoking

## Abstract

**Objectives.** To evaluate whether genetic and environmental factors associated with RA in European and Asian ancestry populations are also associated with RA in African ancestry individuals.

**Methods.** A case–control study was undertaken in 197 RA cases and 868 controls of African ancestry (Black African, Black Caribbean or Black British ethnicity) from South London. Smoking and alcohol consumption data at RA diagnosis was captured. Genotyping was undertaken (Multi-Ethnic Genotyping Array) and human leukocyte antigen (HLA) alleles imputed. The following European/Asian RA susceptibility factors were tested: 99 genome-wide loci combined into a genetic risk score; HLA region [20 haplotypes; shared epitope (SE)]; smoking; and alcohol consumption. The SE was tested for its association with radiological erosions. Logistic regression models were used, including ancestry-informative principal components, to control for admixture.

**Results.** European/Asian susceptibility loci were associated with RA in African ancestry individuals. The genetic risk score provided an odds ratio (OR) for RA of 1.53 (95% CI: 1.31, 1.79; *P *= 1.3 × 10 ^−^^7^). HLA haplotype ORs in European and African ancestry individuals were highly correlated (*r* = 0.83, 95% CI: 0.56, 0.94; *P *= 1.1 × 10 ^−^^4^). Ever-smoking increased (OR = 2.36, 95% CI: 1.46, 3.82; *P* = 4.6 × 10 ^−^^4^) and drinking alcohol reduced (OR = 0.34, 95% CI: 0.20, 0.56; *P *= 2.7 × 10 ^−^^5^) RA risk in African ancestry individuals. The SE was associated with erosions (OR = 2.61, 95% CI: 1.36, 5.01; *P *= 3.9 × 10 ^−^^3^).

**Conclusion.** Gene–environment RA risk factors identified in European/Asian ancestry populations are relevant in African ancestry individuals. As modern statistical methods facilitate analysing ancestrally diverse populations, future genetic studies should incorporate African ancestry individuals to ensure their implications for precision medicine are universally applicable.

Rheumatology key messages

- RA gene–environment risk factors in European and Asian populations are generalizable to African ancestry individuals.
- Smoking and alcohol (dominant European/North-American environmental RA risks) are associated with RA in African ancestry individuals.
- The shared epitope predicts erosive status in African ancestry RA patients.

## Introduction

RA is a complex disease resulting from environmental exposures in genetically predisposed individuals [1]. Many genetic and environmental RA risk factors have been identified in European and Asian ancestry individuals [2–5]. Their generalizability to other ancestral populations is uncertain. The benefits of establishing RA risk factors include facilitating the identification of novel therapeutic targets [5] and risk prediction modelling [6].

Although RA causes significant health-care burdens in Africa [7], few studies have assessed RA risk factors in Africa or African ancestry groups. Relevant Africa-based research comprises several small case–control studies (including <60 cases) [8–10]. Two Cameroonian studies showed the shared epitope (SE) was three times commoner in RA cases [9], but a genetic risk score (GRS) of 28 European RA risk single nucleotide polymorphisms (SNPs) was not associated with RA [8]. A Senegalese study reported increased RA risk in *HLA-DR3* and *HLA-DR10* carriers [10]. RA susceptibility factors in African Americans are better characterized, with smoking and the SE being established risk factors [11]. To date, no studies have examined RA risks in UK-based African ancestry individuals. As UK, USA and Africa-based African ancestry populations are ethnically different (using different self-reported ethnicity classifications), and are likely to differ genetically (with varying degrees of genetic admixture), it is crucial to evaluate RA risks in African ancestry individuals living in the UK.

We evaluated whether gene–environment RA risk factors identified in European and Asian ancestry populations are relevant to African ancestry UK individuals. Our novel case–control study (comprising 197 RA cases and 868 controls of Black African, Black Caribbean and Black British ethnicity) tested whether: the HLA locus, 99 genome-wide loci, smoking, and alcohol consumption were associated with RA in this population. We also compared HLA region contributions to RA risk in African and European ancestry individuals, and evaluated associations between the SE and radiological erosions.

## Methods

### Guidelines

The STrengthening the Reporting of OBservational studies in Epidemiology (STROBE) guidelines have been devised to strengthen transparency in the analysis and reporting of observational studies. We adhered to the STROBE checklist for study reporting [12].

### Subjects

#### African ancestry cohort

African ancestry cases were from the GENetics of RA in individuals of African ancestry (GENRA) study [13]. GENRA recruited 212 patients (January 2011 to February 2015) from rheumatology clinics in four South London hospitals (Guy’s, King’s College, St George’s and Lewisham). Inclusion criteria comprised an RA diagnosis fulfilling the 1987/2010 ACR criteria, and Black African, Black Caribbean or Black British self-reported ethnicity [14]. Patients were assessed once, recording ethnicity, disease characteristics (year diagnosed, medications, serology, extra-articular features) and outcomes (disease activity, disability, quality of life). Patients were asked about current and previous smoking, including time-points for starting/stopping smoking, cigarettes/day (or alternatives) and breaks from smoking. Alcohol intake was assessed by asking if they were current, previous or never drinkers; alcohol intake (units/day) at diagnosis was collected. Hand and feet radiographs were evaluated for erosions; if unavailable, documentation of erosions in the clinical notes was captured. Serum and DNA was extracted from whole blood.

African ancestry controls (*n* = 887) were included from the South London Ethnicity and Stroke Study (SLESS) [15]. Inclusion criteria comprised being of Black Caribbean or Black African ethnicity, and being free of clinical cerebrovascular disease. SLESS control recruitment was by random selection from primary care lists in St George’s, Guy’s and St Thomas’, and King’s College Hospital catchment areas between 1999 and 2012; emailing St George’s University of London/St George’s Hospital staff; and affixing posters publicizing the study in local leisure centres, primary care surgeries, churches and communities centres. Using population controls from the same catchment area as cases reduced selection bias risk. Lifestyle habits were captured by self-completed questionnaire, checked and completed, if necessary, by a clinician interviewing participants. Smoking was assessed by asking: Do you smoke? and Are you an ex-smoker? Alcohol intake was assessed by asking: How many alcohol units do you drink per week?

#### European ancestry cohort

We included European ancestry cases from the Combination Anti-Rheumatic Drugs in Early RA (CARDERA) genetics cohort and controls from the Wellcome Trust Case Control Consortium 2 (WTCCC2) to compare the contribution of the SE to RA risk between African and European ancestry groups. Both cohorts have been described previously [16, 17]. CARDERA comprises 524 patients with early, active RA enrolled in two clinical trials. WTCCC2 controls are from the 1958 Birth Cohort.

#### Genotyping

GENRA and SLESS were genotyped together on the Illumina Multi-Ethnic Genotyping Array, a multi-ethnic platform with >1.7 million markers [18]. Quality control (QC) and imputation procedures are described in supplementary Table S1, available at *Rheumatology* Online. Post-QC 197 cases and 868 controls were available. Principal components (PCs) were calculated, combining GENRA/SLESS with individuals from 1000 Genomes phase 3 populations using smartpca (EIGENSTRAT) on an LD-pruned subset of data [19]. Data were imputed to the 1000 Genomes phase 3 reference set using IMPUTE2 (ver. 2.3.0); 4-digit HLA alleles were imputed using HLA*IMP:02 (multi-ethnic reference panel, imputing 4-digit HLA alleles with 84% accuracy in Africans) [20].

CARDERA cases and WTCCC2 controls were genotyped separately on the Illumina Immunochip [21]. QC was initially performed on each dataset separately and subsequently after merging (using the same procedures as for GENRA/SLESS). PC analysis (PCA) was performed using smartpca (EIGENSTRAT) on an LD-pruned subset [19]. Individuals were removed that were >8 s.d.s from the mean of the first five PCs. Post-QC 520 cases and 2648 controls were available. Imputation of HLA alleles was performed with HLA*IMP:02 (European reference panel, imputing 4-digit HLA alleles with 94% accuracy in Europeans) [20].

### Statistical analysis

#### HLA associations

Raychaudhuri *et al.* [22] showed that most HLA-derived risk for ACPA-positive RA in Europeans is from polymorphisms in five amino acids [22].

These define 16 haplotypes in HLA-DRβ1, 2 haplotypes in HLA-B and 2 haplotypes in HLA-DPβ1. We tested their association with RA in our African and European ancestry cohorts using logistic regression. In GENRA/SLESS, the first 10 ancestry-informative PCs were included as covariates, to adjust for admixture. Pearson’s correlation coefficient tested correlations between the log [odds ratios (ORs)] for association of each HLA haplotype in the meta-analysis, and GENRA/SLESS. As Raychaudhuri *et al.* determined these haplotypes by using omnibus tests to define critical HLA-DRβ1 molecule amino acid positions for RA susceptibility, we additionally used omnibus tests to evaluate the associations between the critical amino acid positions 11, 13, 71 and 74 in HLA-DRβ1 and RA in our African and European ancestry cohorts. We also tested associations between the SE and RA using logistic regression. Nagelkerke’s [23] measure of proportion of trait variance explained compared the degree of RA risk explained by the SE in European and African ancestry individuals.

#### Genome-wide associations

A trans-ethnic RA genome-wide association study (GWAS) meta-analysis by Okada *et al.* [5] identified 102 risk SNPs in European and Asian ancestry individuals. We constructed a genetic risk score (GRS) including 99 of these variants [omitting two X-chromosome markers, and one SNP (rs147622113) not present in Africans] and tested its association with RA in our African ancestry cohort using logistic regression, including the first 10 ancestry-informative PCs. All SNPs included in the GRS had INFO scores >0.55. The GRS was created by summing the number of risk alleles carried at each SNP, weighted by its log (OR). To ensure the GRS association with RA in GENRA/SLESS was not due to European admixture, we divided GENRA cases into quartiles based on PC 1 (separating European and African ancestry individuals) and repeated the GRS analysis for cases in each quartile against all controls. It was not possible to test the association between the GRS and RA in our European ancestry cohort because CARDERA was genotyped on the ImmunoChip, which is missing a substantial proportion of the SNPs identified by Okada *et al.*

#### Smoking and alcohol associations

Smoking and alcohol abstinence have consistent associations with RA in European/North American studies [2, 3]. We tested the association between (i) being an ever-smoker *vs* never-smoker and drinker *vs* non-drinker at RA diagnosis and (ii) case–control status in GENRA/SLESS. GENRA cases were classified as non-drinkers if they reported being a never drinker or if a current/previous drinker reported consuming 0 U/week at RA diagnosis. SLESS controls were classified as non-drinkers if they reported drinking 0 U/week at assessment. Propensity score matching (1:2 ratio) matched GENRA cases with SLESS controls for age, sex and ethnicity. As Black British ethnicity individuals were not recruited to SLESS, 19 Black British GENRA cases were excluded. Eight GENRA cases and 190 SLESS controls with missing smoking/alcohol data were also omitted. Associations between smoking and drinking and RA were tested using multivariate logistic regression models, including age, sex and ethnicity as covariates. Due to ethnic differences in lifestyle habits, a secondary analysis stratifying by ethnicity was performed.

#### Smoking–SE interaction

An additive interaction between the SE and smoking on RA risk is well established in Europeans [24]. We tested this in GENRA/SLESS using logistic regression models incorporating smoking (ever *vs* never-smoking) and the SE (0 *vs* any copies), alongside the first 10 ancestry-informative PCs, age and sex as covariates. The interaction between the SE and smoking was evaluated as the relative excess risk due to interaction, using the approach defined by Rothman and Greenland [25].

#### SE and erosive status

The SE associates with erosions in most RA populations [26]. We tested this in GENRA cases using logistic regression including erosions (present *vs* absent) as the response variable, and the first 10 ancestry-informative PCs, age, sex, ACPA and disease duration as covariates.

#### All cases vs ACPA-positive cases

Gene–environment risk factors for RA have stronger associations with ACPA-positive disease. We therefore analysed all RA and ACPA-positive RA cases separately.

#### Sample size

We had 80% power to detect a common variant [minor allele frequency (MAF) = 30%] with OR >2.10 at genome-wide significance (assuming an additive genetic model and RA prevalence in Africans of 0.75%) [7, 27]. Our study was therefore powered to replicate European HLA haplotype associations. Statistical analyses were performed using R, version 3.2.2.

#### Ethics approval and consent to participate

GENRA (National Research Ethics Service Committee London—Dulwich, reference: 11/LO/1244), SLESS (Wandsworth Local Research Ethics Committee, reference: 05/Q0803/324), CARDERA (National Research Ethics Service Committee East of England—Essex, reference: 11/EE/0544) and WTCCC2 were ethically approved. All participants provided consent according to the Declaration of Helsinki. No additional ethical approval was required for this study.

## Results

### Study characteristics

#### African ancestry cohort

Post-QC, there were 197 GENRA cases and 868 SLESS controls (Table 1). Cases and controls had similar mean ages (56.3 *vs* 58.7 years); more cases were female (83% *vs* 48%). Most cases had established (mean duration 9.3 years) ACPA-positive (78%) RA. Mean disease activity was moderate (DAS28 3.97); 40% were erosive.

**Table 1:** African and European ancestry cohort characteristics

	African Ancestry		European Ancestry	
Characteristic	GENRA cases (*n* = 197)	SLESS controls (*n* = 868)	CARDERA cases (*n* = 520)	WTCCC2 controls (*n* = 2648)
Demographics				
Female, *n* (%)	164 (83.3)	416 (47.9)	355 (68.4)	1278 (48.3)
Age, years	56.3 (14.9)	58.7 (12.0)	54.6 (12.6)	–
RA characteristics				
Disease duration, years	9.3 (10.2)	–	0.28 (0.41)	–
ACPA-positive, *n* (%)	153 (77.7)	–	347 (70.1)	–
RF-positive, *n* (%)	152 (77.2)	–	349 (67.1)	–
DAS28	3.97 (1.59)	–	5.88 (1.28)	–
Erosions, *n* (%)	79 (40.1)	–	–	–
Shared epitope				
0 SE copies, *n* (%)	112 (56.9)	666 (76.8)	135 (25.9)	1302 (49.2)
1 SE copy, *n* (%)	76 (38.6)	192 (22.1)	273 (52.5)	1137 (42.9)
2 SE copies, *n* (%)	9 (4.6)	9 (1.0)	112 (21.5)	209 (7.9)

Data are mean (s.d.) unless otherwise stated. ACPA status missing in 25 CARDERA cases; smoking data missing in 1 GENRA case and 0 SLESS controls; alcohol consumption data missing in 11 GENRA cases and 190 SLESS controls. SE: shared epitope.

**Fig. 1:**
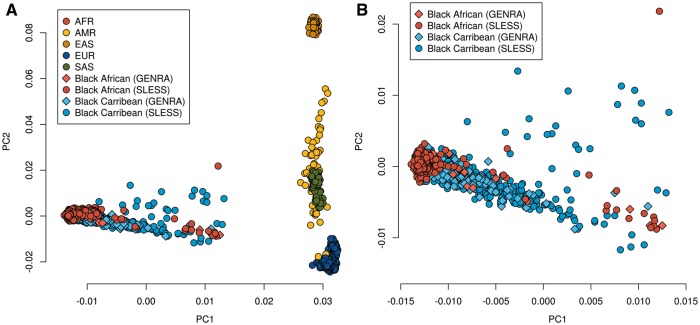
First two ancestry-informative principal components in GENRA, SLESS, and 1000 genomes phase 3 samples (**A**) GENRA, SLESS and 1000 genomes phase 3 samples. (**B**) GENRA and SLESS samples only; (**B**) is an enlarged representation of the results presented in (**A**), without including the 1000 genomes phase 3 samples. AFR: African; AMR: Admixed American; EAS: East Asian; EUR: European; SAS: South Asian.

PCA showed strong segregation of GENRA/SLESS samples with African populations from 1000 Genomes phase 3 (Fig. 1). While European admixture in GENRA and SLESS was observed, this was not significantly different between them. The major PC dividing European from African ancestry (PC1) was not significantly associated with RA status under logistic regression (*P* = 0.27).

#### European ancestry cohort

Post-QC, there were 520 CARDERA cases and 2648 WTCCC2 controls. More cases were female (68% *vs* 48%). Cases had early, active disease at trial baseline (mean duration 0.28 years; mean DAS28 5.88). Most were ACPA-positive (70%).

#### HLA imputation

HLA alleles were imputed with a high-degree of certainty (supplementary Figs S1–4, available at *Rheumatology* Online). In GENRA/SLESS, 232 alleles were imputed at 4-digit resolution; 127 alleles had a frequency of >1%. The median posterior probability (Q) of allele allocation was 0.974; 88.1% of alleles were imputed with Q > 0.9; 96.3% were imputed with Q > 0.8. In CARDERA/WTCCC2, 183 alleles were imputed at 4-digit resolution; 111 of these had a frequency of >1%. The median Q of allele allocation was 0.975; 87.3% of alleles were imputed with Q > 0.9; 96.8% were imputed with Q > 0.8. Lower frequency alleles were imputed with less certainty, as expected due to their rarity in reference panels. Imputation posterior probabilities for the HLA region were in general similar between RA cases and controls, with no significant differences between them as assessed by linear regression. The exception was for HLA-DPB1, which showed slightly lower imputation quality in controls compared with cases (*P *= 0.024).

#### HLA associations with RA in African and European ancestry cohorts

**Fig. 2:**
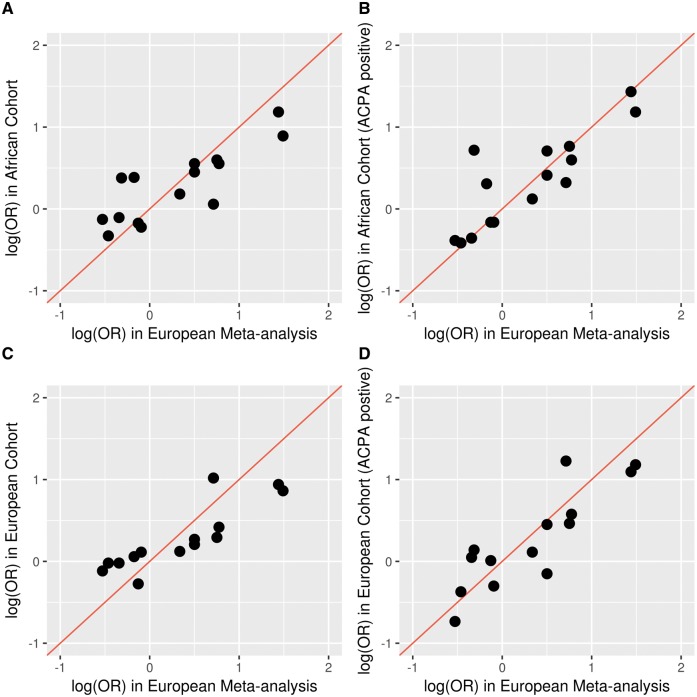
RA Odds ratios for HLA haplotypes in the African/European studies *vs* the European meta-analysis (**A**) All African ancestry RA cases. (**B**) ACPA-positive African ancestry RA cases. (**C**) All European ancestry RA cases. (**D**) ACPA-positive European ancestry RA cases. All European ancestry cases in meta-analysis were ACPA-positive; reference haplotypes [i.e. those with log (OR) = 0] not plotted. African study: GENRA/SLESS; European study: CARDERA/WTCCC2; European meta-analysis is the study by Raychaudhuri *et al.*

HLA haplotypes had similar effects on RA risk in GENRA/SLESS to those observed in the European meta-analysis (Table 2). Using the commonest control haplotype in the meta-analysis as the reference haplotype, in GENRA/SLESS only 2 (from 15 available non-reference haplotypes) had ORs in opposing directions to the European meta-analysis. In CARDERA/WTCCC2, 4 (from 16 available non-reference haplotypes) had ORs in opposing directions to the European meta-analysis. Haplotype ORs in GENRA/SLESS and the European meta-analysis were highly correlated (*r* = 0.83, 95% CI: 0.56, 0.94; *P *= 1.1 × 10 ^−^^4^; Fig. 2). In GENRA/SLESS one further haplotype was observed, which was not present in Europeans [encoded by HLA-DRB1*14:04; MAF = 0.005; OR = 0.96].

**Table 2:** HLA haplotype associations with ACPA-positive RA in European meta-analysis, compared with African and European Studies

HLA-DRβ1 amino acids at position				Classical *HLA-DRB1* alleles	Meta-analysis	European cohort	African cohort
11	13	71	74		OR (Freq Ca, Co)	OR (Freq Ca, Co)	OR (Freq Ca, Co)
Val	His	Lys	Ala	*04:01	4.44 (0.316, 0.106)	3.26 (0.227, 0.120)	3.27 (0.028, 0.014)
Val	His, Phe	Arg	Ala	*04:08, *04:05,*04:04, *10:01	4.22 (0.141, 0.056)	2.99 (0.113, 0.056)	4.19 (0.107, 0.039)
Leu	Phe	Arg	Ala	*01:02, *01:01	2.17 (0.143, 0.109)	1.78 (0.138, 0.117)	1.82 (0.104, 0.068)
Pro	Arg	Arg	Ala	*16:01**,** *16:02	2.04 (0.012, 0.013)	3.41 (0.011, 0.005)	1.38 (0.018, 0.020)
Val	His	Arg	Glu	*04:03, *04:07, *04:11	1.65 (0.009, 0.010)	0.86 (0.010, 0.010)	2.03 (0.005, 0.003)
Asp	Phe	Arg	Glu	*09:01	1.65 (0.013, 0.011)	1.57 (0.012, 0.011)	1.51 (0.050, 0.034)
Val	His	Glu	Ala	*04:02	1.43 (0.006, 0.011)	–	– (0.008, –)
Ser	Ser	Lys	Ala	*13:03	1.04 (0.006, 0.012)	1.02 (0.009, 0.009)	– (0.046, –)
Pro	Arg	Ala	Ala	*15:01, *15:02, *15:03	1.00 (0.092, 0.142)	1.00 (0.118, 0.144)	1.00 (0.147, 0.162)
Gly	Tyr	Arg	Gln	*07:01	0.91 (0.064, 0.133)	0.74 (0.090, 0.147)	0.85 (0.063, 0.088)
Ser	Ser, Gly	Arg	Ala	*11:01, *11:04, *11:10, *12:01, *12:02, *14:02	0.88 (0.049, 0.103)	1.01 (0.087, 0.108)	0.85 (0.129, 0.154)
Ser	Ser	Arg	Glu	*14:01	0.84 (0.012, 0.025)	0.33 (0.013, 0.028)	1.36 (0.013, 0.017)
Ser	Gly	Arg	Glu	*14:04	–	–	0.96 (0.005, 0.005)
Leu	Phe	Glu	Ala	*01:03	0.73 (0.002, 0.004)	1.15 (0.004, 0.004)	2.05 (0.003, 0.002)
Ser	Gly	Arg	Leu	*08:01, *08:02, *08:04, *08:06	0.71 (0.013, 0.028)	1.05 (0.019, 0.024)	0.70 (0.069, 0.083)
Ser	Ser	Lys	Arg	*03:01, *03:02	0.63 (0.083, 0.128)	0.69 (0.118, 0.143)	0.66 (0.089, 0.134)
Ser	Ser	Glu	Ala	*11:02, *11:03, *13:01, *13:02, *13:04	0.59 (0.041, 0.112)	0.48 (0.044, 0.099)	0.68 (0.124, 0.152)
HLA-B amino acid at position 9				Classical *HLA-B* alleles			
Asp				*08	2.12 (0.130, 0.118)	1.59 (0.137, 0.141)	2.15 (0.046, 0.029)
His, Tyr				*07, *13, *14, *15, *18, *27, *35, *37, *38, *39, *40, *41, *44, *45, *47, *49, *50, *51, *52, *53, *55, *56, *57, *58, *73	1.00 (0.870, 0.882)	1.00 (0.863, 0.859)	1.00 (0.954, 0.971)
HLA-DPβ1 amino acid at position 9				Classical *HLA-DPB1* alleles			
Phe				*02:01, *02:02, *04:01, *04:02, *05:01, *16:01, *19:01, *23:01	1.40 (0.799, 0.728)	1.12 (0.727, 0.689)	1.13 (0.353, 0.318)
His, Tyr				*01:01, *03:01, *06:01, *09:01, *10:01, *11:01, *13:01, *14:01, *15:01, *17:01, *20:01	1.00 (0.201, 0.272)	1.00 (0.273, 0.311)	1.00 (0.647, 0.682)

Reference haplotype is the commonest one observed in the meta-analysis controls; classical *HLA-DRB1* SE alleles are shown in bold. European meta-analysis by Raychaudhuri *et al.*; African cohort: GENRA/SLESS; European cohort: CARDERA/WTCCC2. Freq: allele frequency in cases (Ca) or controls (Co); – : haplotype either not present or not imputed to sufficient accuracy.

Omnibus tests showed highly significant associations for amino acid positions 11 and 13 in HLA-DRβ1 (*P *= 8.1 × 10 ^−^^9^, *P *= 7.8 × 10 ^−^^9^, respectively) and case–control status in GENRA/SLESS, but not positions 74 (*P = *0.017) and 71 (*P = *0.20). In CARDERA/WTCCC2, highly significant associations were observed for amino acids positions 11, 13 and 71 in HLA-DRβ1 (*P *= 1.6 × 10 ^−^^10^, *P *= 1.2 × 10 ^−^^10^ and *P *= 5.0 × 10 ^−^^9^, respectively) with case–control status. The association at position 74 was not significant (*P *= 0.058).

SE alleles were rarer in African than European ancestry individuals (Tables 1 and 3). The data indicated a SE frequency of 24% and 12% in GENRA cases and SLESS controls, respectively, and 48% and 29% in CARDERA cases and WTCCC2 controls, respectively. The OR for RA per SE copy was similar in African (OR = 2.41, 95% CI: 1.80, 3.23; *P *= 4.6 × 10 ^−^^9^) and European (OR = 2.27, 95% CI: 2.05, 2.51; *P *= 1.6 × 10 ^−^^57^) ancestry cohorts. The proportion of variance in case–control status explained by the SE was also similar (African *R*^2 ^=0.050; European *R*^2 ^=0.069). The SE was commoner in ACPA-positive cases, with larger effects on risk observed (Table 3).

**Table 3:** Shared epitope impact on RA risk in African and European ancestry cohorts

SE freq and association with RA	European cohort	African cohort
All RA		
SE freq (cases)	0.48	0.24
SE freq (controls)	0.29	0.12
OR (95% CI)	2.27 (2.05, 2.51)	2.41 (1.80, 3.23)
*P*-value	1.6 × 10^−57^	4.6 × 10^−9^
*R*^2^	0.069	0.050
ACPA + RA		
SE freq (cases)	0.54	0.28
SE freq (controls)	0.29	0.12
OR (95% CI)	3.03 (2.68, 3.42)	3.03 (2.20, 4.20)
*P*-value	2.9 × 10^−71^	7.9 × 10^−12^
*R*^2^	0.107	0.079

*R*^2 ^: Nagelkerke’s measure of proportion of trait variance explained. Freq: SE allele frequency; OR: odds ratio per copy of SE allele carried in additive model (relative to no SE copies).

#### Genome-wide SNP associations with RA in African ancestry individuals

The GRS was significantly associated with RA in GENRA/SLESS (OR = 1.53, 95% CI: 1.31, 1.79; *P* = 1.3 × 10 ^−^^7^). Although the HLA SNP included in the GRS contributed a substantial part of this signal, a significant association remained when it was omitted (OR = 1.30, 95% CI: 1.11, 1.52; *P* = 0.0011). The association was stronger when restricting to ACPA-positive cases (OR = 1.75, 95% CI: 1.47, 2.08; *P *= 5.0 × 10 ^−^^10^); this remained significant on omitting the HLA SNP (OR = 1.40, 95% CI: 1.18, 1.67; *P *= 1.7 × 10 ^−^^4^). Individual SNP associations are provided in supplementary Table S2, available at *Rheumatology* Online.

Dividing GENRA cases into quartiles based on their proportion of European ancestry (captured by the first PC) and repeating the GRS analysis for each quartile against all controls ensured the association was not driven by European admixture (supplementary Fig. S5, available at *Rheumatology* Online).

Plotting the ORs for RA associated with each SNP in the Okada *et al.* meta-analysis and our GENRA/SLESS African ancestry cohort and scaling each point by their minor allele frequency (supplementary Fig. S6, available at *Rheumatology* Online) showed that the majority of common RA susceptibility SNPs share a direction of effect in the meta-analysis and our African ancestry cohort. Although there are some SNPs that have an opposite direction of effect, these are generally rare. Overall 56/99 (57%) of SNPs share a direction of effect between the Okada *et al.* meta-analysis and our African ancestry cohort. Stratifying this analysis by MAF shows that for markers with MAF > 0.01, 52/90 (58%) share a direction of effect; for markers with MAF > 0.05, 49/78 (63%) share a direction of effect; and for markers with MAF > 0.1, 44/64 (69%) share a direction of effect.

#### Association between smoking, alcohol and RA in African ancestry individuals

Smoking was associated with RA in GENRA/SLESS (Table 4). The OR for RA in ever *vs* never-smokers was 2.36 (95% CI: 1.46, 3.82; *P *= 4.6 × 10 ^−^^4^). Similar results were observed for ACPA-positive cases (OR = 2.13, 95% CI: 1.24, 3.66; *P = *6.0 × 10 ^−^^3^; supplementary Table S3, available at *Rheumatology* Online). Univariate estimates remained significant (supplementary Table S4, available at *Rheumatology* Online). There was no evidence of an interaction between the SE and smoking on RA risk, when evaluated on the additive scale (OR = 3.15, 95% CI: 0.25, 39.9).

**Table 4:** Association between smoking, alcohol and RA in African ancestry cases and controls

Characteristic	GENRA cases	SLESS controls	OR (95% CI)	*P*-value
Black Caribbean and Black African individuals (GENRA, *n* = 170; SLESS, *n* = 340)				
Age, mean (s.d.), years	57.2 (14.9)	58.9 (11.8)	0.99 (0.97, 1.00)	0.11
Female, *n* (%)	141 (82.9)	282 (82.9)	1.09 (0.61, 1.94)	0.77
Caribbean ethnicity, *n* (%)	104 (61.1)	217 (63.8)	0.88 (0.58, 1.34)	0.56
Ever-smoker, *n* (%)	54 (31.8)	59 (20.3)	2.36 (1.46, 3.82)	4.6 × 10^−4^
Alcohol drinker, *n* (%)	28 (16.5)	108 (31.8)	0.34 (0.20, 0.56)	2.7 × 10^−5^
Black Caribbean individuals (GENRA, n = 104; SLESS, n = 217)				
Ever-smoker, *n* (%)	44 (42.3)	55 (25.3)	2.54 (1.46, 4.43)	9.7 × 10^−4^
Alcohol drinker, *n* (%)	20 (19.2)	76 (35.0)	0.33 (0.18, 0.61)	4.4 × 10^−4^
Black African individuals (GENRA, *n* = 66; SLESS, *n* = 123)				
Ever-smoker, *n* (%)	10 (15.2)	14 (11.4)	2.86 (0.96, 8.52)	0.059
Alcohol drinker, *n* (%)	8 (12.1)	32 (26.0)	0.35 (0.14, 0.87)	0.025

Cases and controls matched for age, sex and ethnicity. ORs for RA associated with smoking are adjusted for age, gender and drinking status (and ethnicity in analysis of all individuals); ORs for RA associated with drinking are adjusted for age, gender and smoking status (and ethnicity in analysis of all individuals); unadjusted ORs are given in supplementary Table S4, available at *Rheumatology* Online.

Alcohol consumption had a significant inverse relationship with RA in GENRA/SLESS. The OR for RA in drinkers *vs* non-drinkers was 0.34 (95% CI: 0.20, 0.56; *P *= 2.7 × 10 ^−^^5^). The association was marginally stronger for ACPA-positive RA (OR = 0.30, 95% CI: 0.17, 0.53; *P *= 3.9 × 10 ^−^^5^; supplementary Table 3, available at *Rheumatology* Online). While smoking and drinking rates were lower in Black African individuals compared with Black Caribbean individuals, ORs for RA associated with smoking and alcohol were similar in these ethnic groups (Table 4).

#### SE and erosive status in African ancestry cases

The SE was significantly associated with erosions in GENRA. The OR for RA per SE copy was 2.61 (95% CI: 1.36, 5.01; *P* = 3.9 × 10 ^−^^3^) in all RA cases, and 3.35 (95% CI: 1.64, 6.91; *P *= 1.0 × 10 ^−^^3^) in ACPA-positive cases.

## Discussion

We studied whether gene–environment RA risk factors identified in European and Asian ancestry populations are relevant in UK-based African ancestry individuals. Our study has three key findings. First, we showed that European–Asian RA susceptibility loci (a 99 SNP GRS and 20 HLA haplotypes) are associated with RA in African ancestry individuals. Second, we found that smoking and alcohol (dominant European/North American environmental RA risks [2, 3]) are associated with RA in African ancestry individuals. Third, we demonstrated that the SE, which predicts radiological damage in a range of ancestral groups [26] also predicts erosive status in African ancestry RA patients. Overall, our findings provide strong evidence for a shared genetic–environmental architecture for RA across European, Asian and African ancestry populations.

We used three approaches to examine the association between the HLA locus and RA in GENRA/SLESS. First, we used the 20 haplotype model proposed by Raychaudhuri *et al.* [22]; second, we used omnibus tests to evaluate the critical amino acid positions in HLA-DRβ1 identified by Raychaudhuri *et al.*; and third, we used the SE. In all three instances, the HLA region had a highly significant association with RA. While amino acid position 71 was not associated with RA in GENRA/SLESS, the relevance of this is uncertain, as the SE (which spans positions 71–74) had a highly significant association with RA status. Although SE alleles were rarer in Africans than Europeans, the difference in the proportion of SE allele carriers between cases and controls was similar in both GENRA/SLESS and CARDERA/WTCCC2, resulting in similar ORs for RA. Additionally, the proportion of variance in case–control status explained by the SE was similar, albeit slightly lower in GENRA/SLESS (*R*^2 ^=0.050) compared with CARDERA/WTCCC2 (*R*^2 ^=0.069). This suggests that the HLA region has a comparable impact on RA susceptibility in European and African ancestry groups.

Our study lacked the power to detect individual SNP associations with RA in GENRA/SLESS. We therefore tested a GRS combining validated RA susceptibility SNPs. This approach is widely used to replicate genetic risks in polygenic disorders, whose genetic architecture comprises hundreds to thousands of very small effect common alleles [28, 29]. Excluding the HLA-tagging SNP from our GRS reduced the significance of the association, suggesting that as in Europeans, most of RA’s heritability is from the HLA locus. It is improbable that all validated susceptibility SNPs reported by Okada *et al.* would replicate in a similarly sized African ancestry cohort, but our analysis supports the concept of an overall shared burden of genetic RA risk loci across ancestral groups.

Smoking and abstinence from alcohol increased RA risk in African ancestry individuals, replicating the effects observed in Europeans. While we observed larger ORs for RA associated with these factors compared with the pooled study risks observed in published meta-analyses—meta-analysis OR for RA in drinkers *vs* non-drinkers of 0.78 (95% CI: 0.63, 0.96) [2] and ever- *vs* never smokers of 1.40 (95% CI: 1.25, 1.58) [3]—our modest sample size limited our precision in estimating risk. The ethnic differences we observed in alcohol and smoking habits—with smoking and drinking being commoner in Black Caribbean, compared with Black African individuals—highlight the importance of considering ethnicity when evaluating lifestyle factors, although the small sample sizes of these ethnic subgroups means this finding requires interpreting with caution.

This is the first analysis of associations between erosions and the SE in UK-based African ancestry individuals. The OR of 2.60 in GENRA was similar to a meta-analysis of eight studies containing 532 Northern European RA patients, reporting an OR for RA with one SE allele of 2.4 [26]. In GENRA the association appeared independent of ACPA, which was included as a modelling covariate. More recently we, along with other research groups, have demonstrated a significant association between the presence of Valine at position 11 (external to the SE) in HLA-DRβ1 and radiological damage in European ancestry RA patients [17, 30]. Testing this position in GENRA also revealed a significant association with erosions (OR = 2.15, 95% CI: 1.02, 4.53; *P *= 0.047), although we could not confirm this was independent of the SE owing to our limited sample size.

Our study has several strengths. First, it is the first analysis of RA risk factors in Black British and Black Caribbean individuals. Second, we evaluated a broad range of genetic–environmental factors. Third, pooling African ancestry ethnic groups and using computational techniques to account for population stratification optimized study power. Fourth, population controls were used from the same catchment area as cases. It also has limitations. First, the absence of HLA allele genotyping prevented imputation internal validation; however, posterior-probability scores suggested common HLA variants were accurately imputed: HLA*IMP:02 has documented accuracy at imputing HLA alleles [20], and the SE prevalence in GENRA (24%) was similar to that observed in Cameroonian RA cases (30%) [9]. Second, as RA patients retrospectively recalled lifestyle habits at diagnosis, recall bias was possible. In the context of alcohol, this could inflate the effect seen (RA patients often abstain from alcohol with DMARD use, which could make them more likely to classify themselves as non-drinkers at diagnosis). Third, different methods were used to evaluate lifestyle habits in cases and controls. Finally, controls were not screened for RA; however, its low prevalence (0.6–0.9% in Africans [7]) suggests few would have this disease, and it is not routine practice to screen controls for disease in genetic studies of low prevalence disorders.

Despite increasing UK ethnic diversity (26–31% of our local South London community are of Black ethnicity [31, 32]), few ethnic minority patients participate in research. This issue is particularly pressing in genetic studies, as GWAS traditionally exclude non-Europeans to minimize population stratification. This calls into question the generalizability of research findings to ethnic minority groups. As statistical advances facilitate genomic analyses of ancestrally diverse populations [33, 34], we undertook a proof-of-concept GWAS (including the first 10 ancestry-informative PCs) in our ancestrally heterogeneous GENRA/SLESS cohort (supplementary Figs S7 and S8, available at *Rheumatology* Online). The genomic-inflation factor was 0.99, suggesting appropriate control for population stratification. We therefore propose that future GWAS meta-analyses include African ancestry samples.

In conclusion, genetic and environmental factors for RA susceptibility and severity in European and Asian ancestry populations are generalizable to African ancestry individuals. Greater efforts are needed to include ethnic minorities in research. This will ensure research findings and their potential for clinical benefit (which in the context of susceptibility loci and prognostic markers includes prevention and precision medicine) are universally translatable.

## Supplementary Material

Supplementary Data
