# Supplementary material for: Genetic and environmental risk factors for rheumatoid arthritis in a UK African ancestry population: the GENRA case–control study
Source: Rheumatology (Oxford). 2017 Apr 12;56(8):1282–92. doi: 10.1093/rheumatology/kex048 (PMC5638023; doi:10.1093/rheumatology/kex048)
Supplement: Supplementary Data [file kex048_Supp.docx]

**SUPPLEMENTARY DATA**

**Supplementary Figure S1. Posterior Probabilities (Q) for Imputation of HLA alleles in European Ancestry samples from CARDERA and WTCCC2**

**
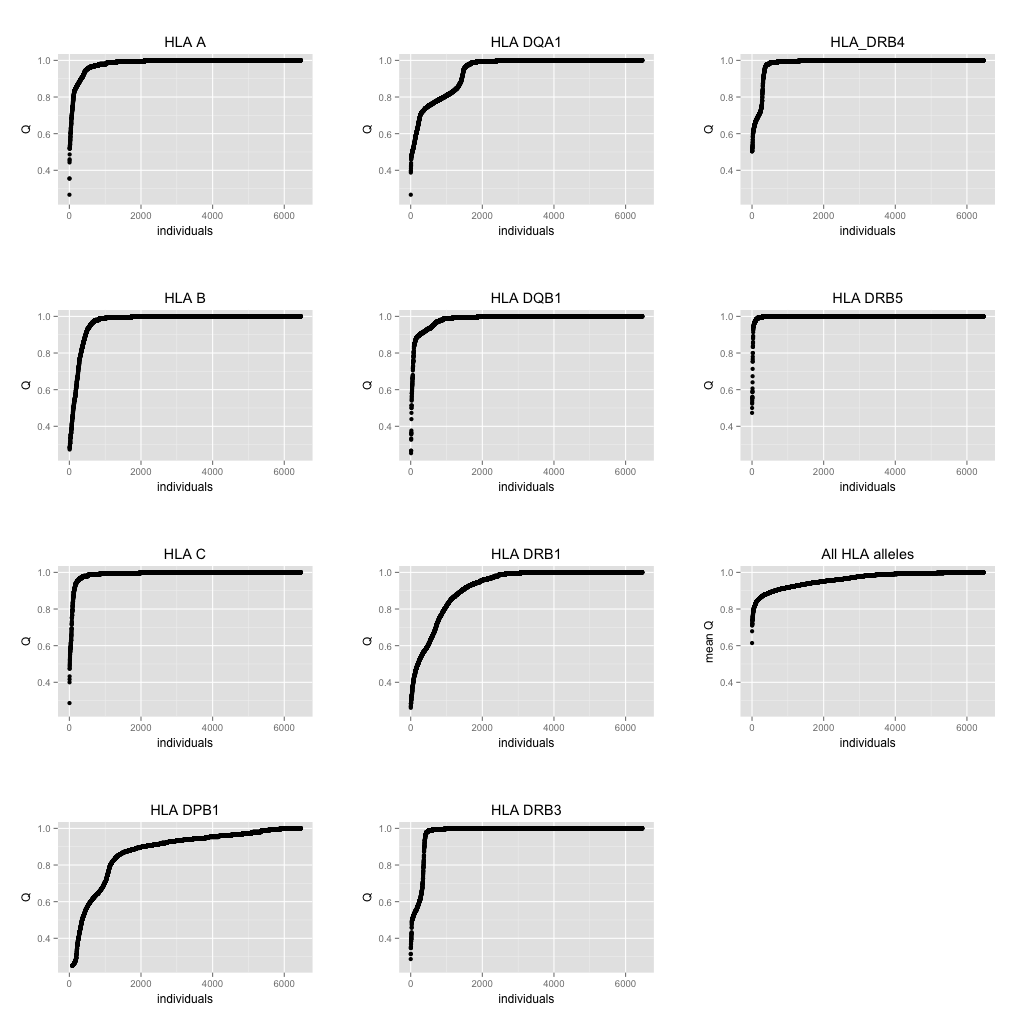
**

**
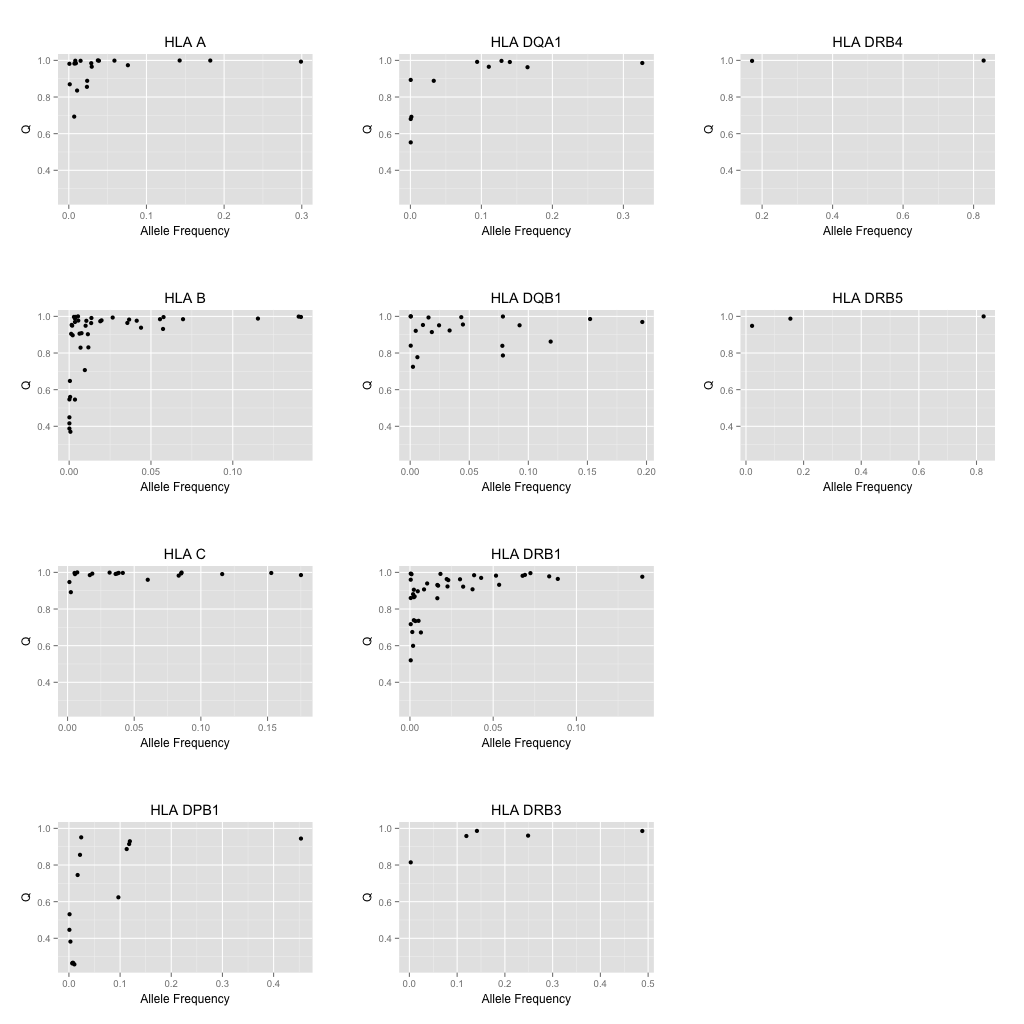
Supplementary Figure S2. HLA Imputation Posterior Probabilities (Q) by Allele Frequency in European Ancestry samples from CARDERA and WTCCC2**


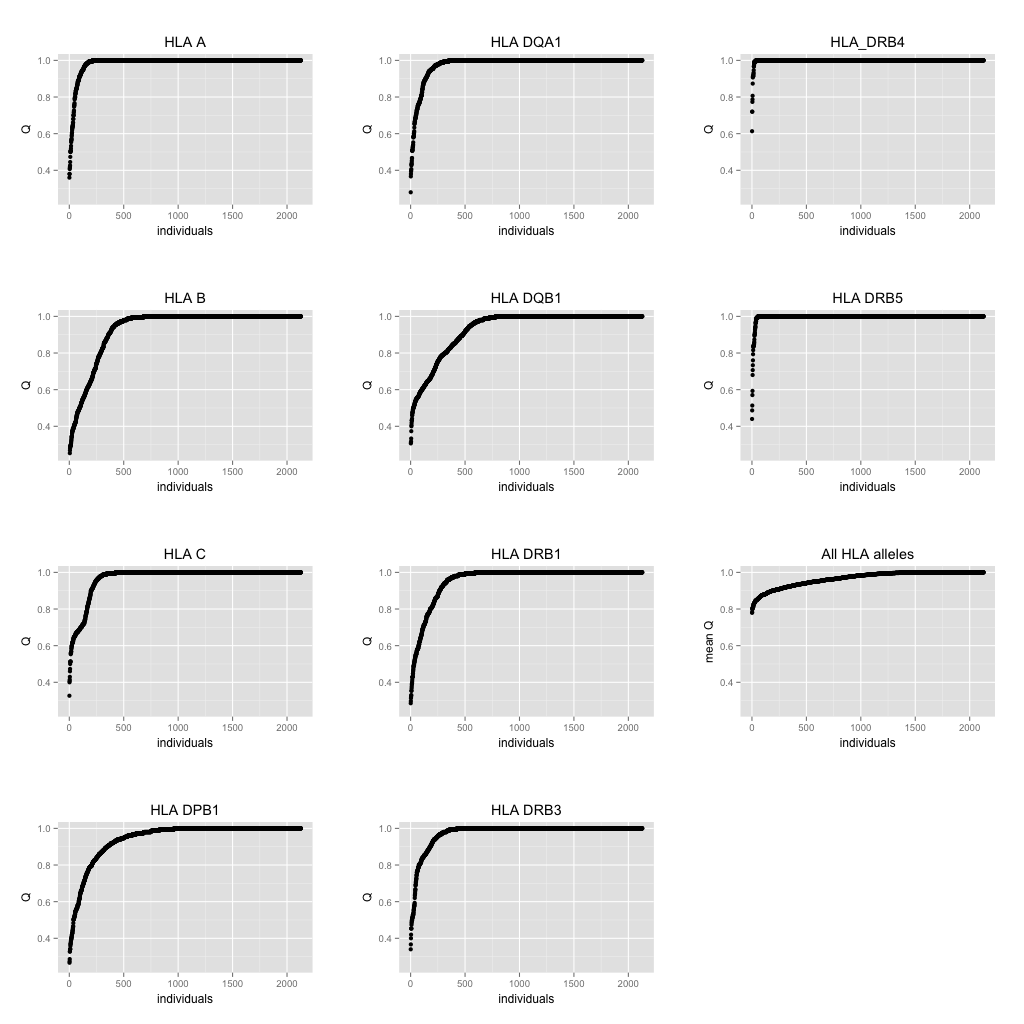
**Supplementary Figure S3. Posterior Probabilities (Q) for Imputation of HLA Alleles in African ancestry samples from GENRA and SLESS**

**
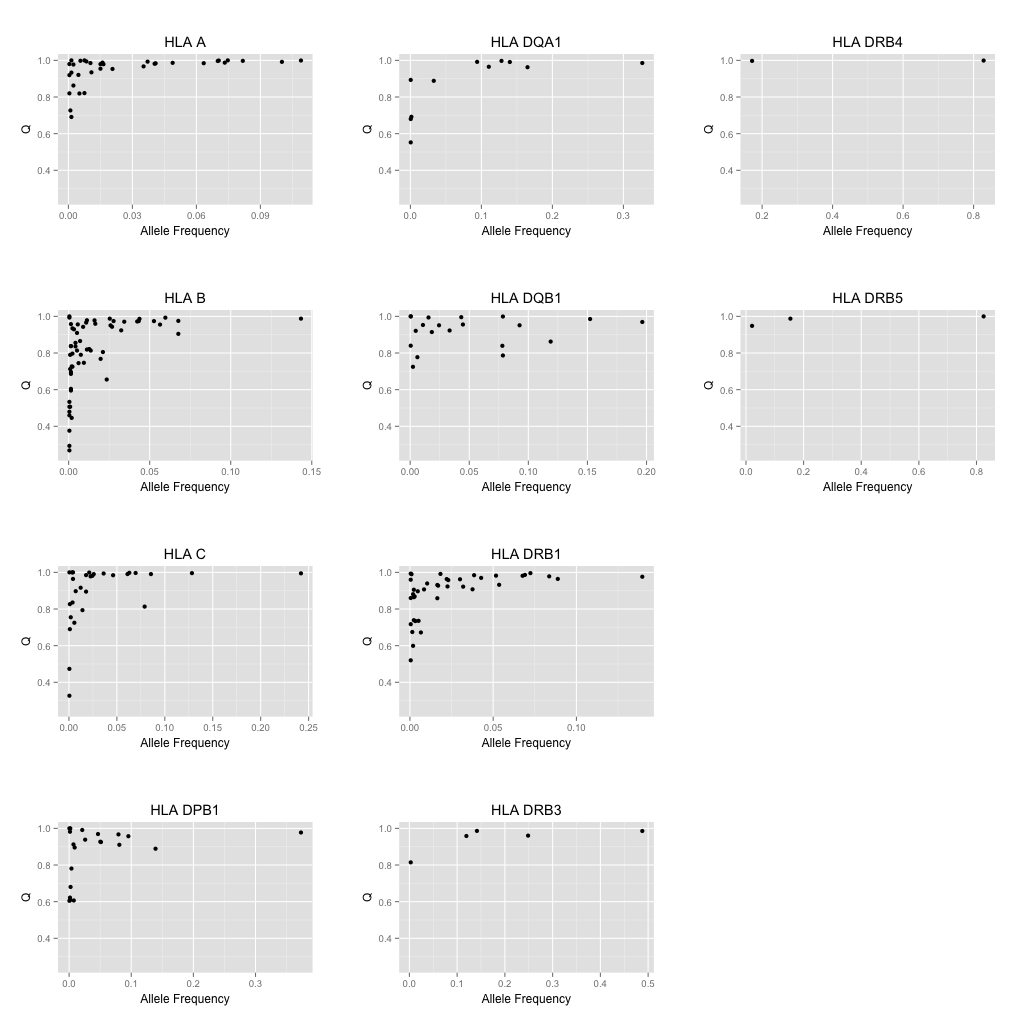
Supplementary Figure S4. HLA Imputation Posterior Probabilities (Q) by Allele Frequency in African ancestry samples from GENRA and SLESS**

**Supplementary Figure S5. Odds Ratios for Association of Genetic Risk Score with RA by Extent of African Ancestry**


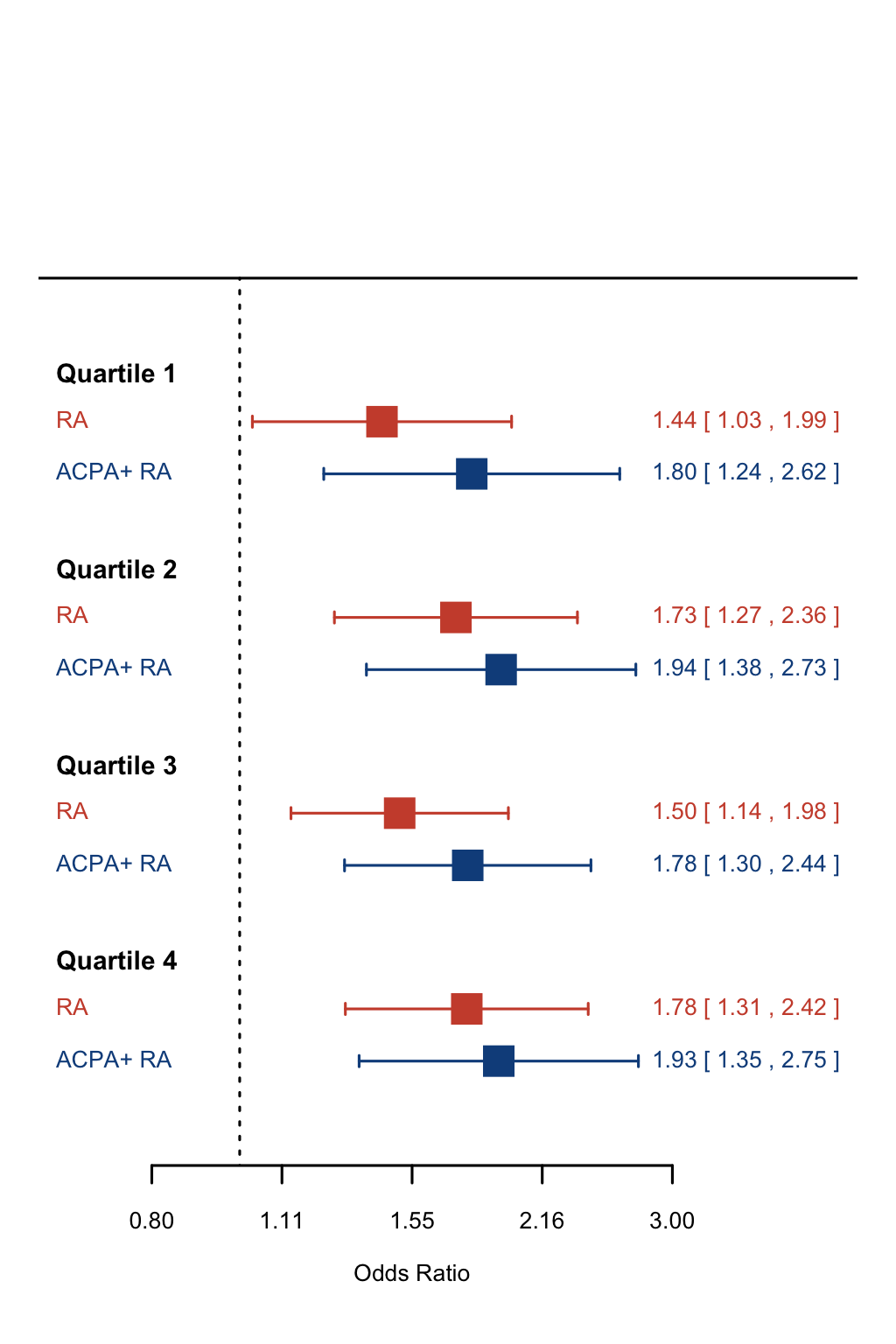


Quartile 1: Most African; Quartile 4: Least African.

.

**Supplementary Figure S6. Odds Ratios for Susceptibility SNPs between GENRA/SLESS and Trans-Ethnic Meta-Analysis by Minor Allele Frequency**

**
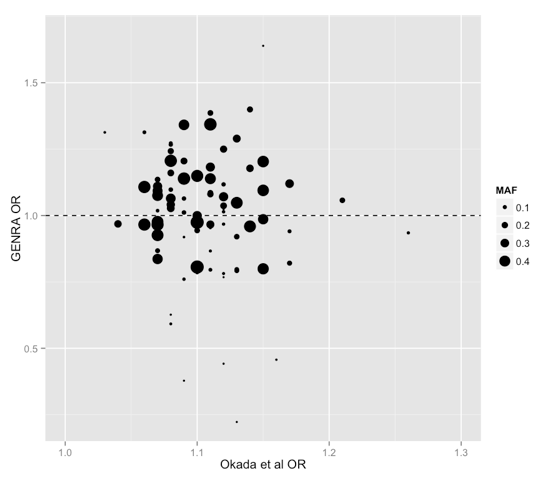
**

Trans-ethnic meta-analysis is the study by Okada et al.

**Supplementary Figure S7. Manhattan Plot for Association of Genome-Wide SNPs with RA in African Ancestry Cases/Controls
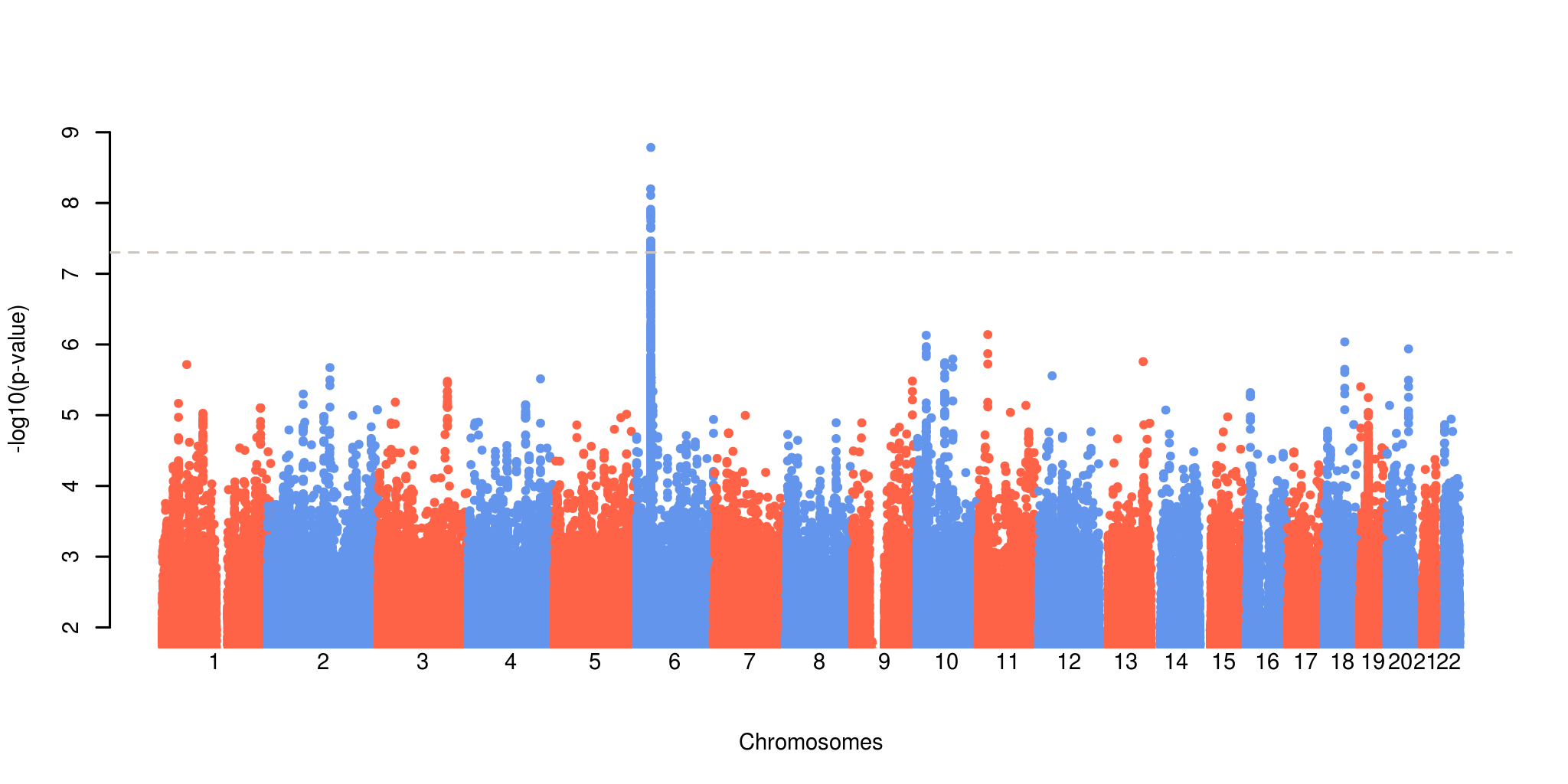
**

SNPs: single nucleotide polymorphisms.

**Supplementary Figure S8. Quantile-Quantile Plot of Observed versus Expected Genome-Wide Association Statistics in African Ancestry Cases/Controls**


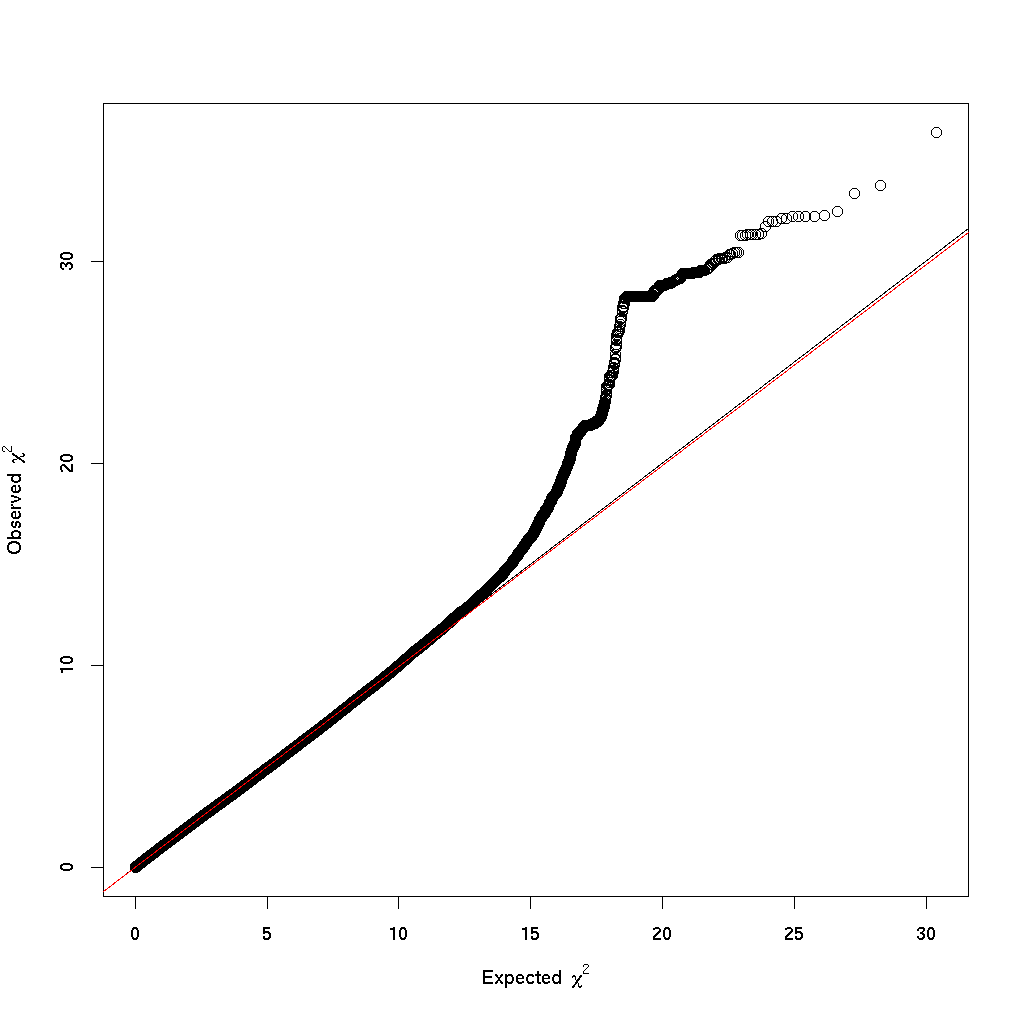


λ = 0.99

**Supplementary Table S1. Quality Control and Imputation Procedures in African Ancestry Cohort (GENRA and SLESS)**

|  | **Description** |
| --- | --- |
| **Quality Control** | SNPs were removed that showed excess missingness (>3%), deviation from Hardy-Weinberg equilibrium (HWE; *P*<x10^-6^) or low minor allele frequency (MAF <0.005). Individuals with excess missingness (>3%), low or high levels of heterozygosity, relatedness (pi-hat>0.1875) and discordant phenotypic/genotypic gender information were removed (15 cases and 19 controls). |
| **Imputation** | Data were imputed to 1,000 Genomes Phase 3 reference set using SHAPEIT (v2.r778) for phasing and IMPUTE2 (v2.3.0) for imputation. Prior to imputation, strand ambiguous SNPs (A/G and C/T) were removed. A total of 821,766 SNPs were used for imputation. Imputation of four-digit HLA alleles was performed with HLA*IMP:02, using a multi-ethnic reference panel comprising African, European, Hispanic, and Asian ancestry individuals. |

**Supplementary Table S2. Association of Autosomal RA-Associated SNPs from Trans-ethnic meta-analysis with RA in GENRA/SLESS**

| **SNP** | **CHR** | **BP** | **A1** | **A2** | **FRQ** | **OR** | **SE** | ***P*-Value** |
| --- | --- | --- | --- | --- | --- | --- | --- | --- |
| rs9268839 | 6 | 32428772 | A | G | 0.735 | 0.595 | 0.121 | 0.000018 |
| rs6479800:64036881:G:C | 10 | 64036881 | G | C | 0.469 | 0.744 | 0.114 | 0.010 |
| rs4452313:17047032:A:T | 3 | 17047032 | A | T | 0.388 | 0.746 | 0.119 | 0.014 |
| rs2736337 | 8 | 11341880 | T | C | 0.826 | 0.721 | 0.141 | 0.021 |
| rs3087243 | 2 | 204738919 | G | A | 0.814 | 1.400 | 0.161 | 0.037 |
| rs34536443:10463118:G:C | 19 | 10463118 | G | C | 0.999 | 0.009 | 2.371 | 0.049 |
| rs998731 | 8 | 81095395 | C | T | 0.045 | 1.690 | 0.267 | 0.050 |
| rs11933540 | 4 | 26120001 | T | C | 0.416 | 1.251 | 0.115 | 0.051 |
| rs8032939 | 15 | 38834033 | T | C | 0.268 | 0.775 | 0.135 | 0.059 |
| rs331463:36501787:A:T | 11 | 36501787 | A | T | 0.501 | 1.241 | 0.116 | 0.063 |
| rs1980422 | 2 | 204610396 | C | T | 0.236 | 1.250 | 0.126 | 0.076 |
| rs147868091:35928240:C:T | 21 | 35928240 | C | T | 0.874 | 1.350 | 0.183 | 0.101 |
| rs8026898 | 15 | 69991417 | G | A | 0.429 | 0.831 | 0.115 | 0.109 |
| rs11089637 | 22 | 21979096 | T | C | 0.540 | 0.829 | 0.117 | 0.109 |
| rs2671692 | 10 | 50097819 | G | A | 0.626 | 1.196 | 0.119 | 0.133 |
| rs71508903 | 10 | 63779871 | C | T | 0.979 | 2.188 | 0.536 | 0.145 |
| rs74984480:14103212:C:T | 6 | 14103212 | C | T | 0.990 | 4.495 | 1.065 | 0.159 |
| rs3824660:8104722:C:T | 10 | 8104722 | C | T | 0.808 | 1.243 | 0.156 | 0.164 |
| rs73013527 | 11 | 128496952 | C | T | 0.899 | 1.330 | 0.206 | 0.165 |
| rs947474 | 10 | 6390450 | G | A | 0.322 | 0.846 | 0.121 | 0.166 |
| rs4239702 | 20 | 44749251 | T | C | 0.131 | 1.263 | 0.169 | 0.167 |
| rs1877030 | 17 | 37740161 | T | C | 0.223 | 0.830 | 0.137 | 0.173 |
| rs793108 | 10 | 31415106 | C | T | 0.892 | 0.789 | 0.175 | 0.176 |
| rs2582532 | 14 | 105392837 | T | C | 0.143 | 1.219 | 0.149 | 0.184 |
| rs657075 | 5 | 131430118 | G | A | 0.987 | 2.646 | 0.739 | 0.188 |
| rs4409785 | 11 | 95311422 | T | C | 0.983 | 2.263 | 0.625 | 0.192 |
| rs2561477 | 5 | 102608924 | G | A | 0.930 | 0.760 | 0.214 | 0.200 |
| rs11889341 | 2 | 191943742 | C | T | 0.875 | 1.255 | 0.182 | 0.212 |
| rs67250450 | 7 | 28174986 | T | C | 0.548 | 1.149 | 0.114 | 0.223 |
| rs10790268:118729391:A:G | 11 | 118729391 | A | G | 0.251 | 0.849 | 0.135 | 0.226 |
| rs1858037:65598300:T:A | 2 | 65598300 | T | A | 0.914 | 0.796 | 0.195 | 0.241 |
| rs2451258:159506600:C:T | 6 | 159506600 | C | T | 0.079 | 1.273 | 0.214 | 0.260 |
| rs10028001:79502972:T:A | 4 | 79502972 | T | A | 0.912 | 1.314 | 0.242 | 0.260 |
| 12:56394954:A:G | 12 | 56394954 | A | G | 0.534 | 1.139 | 0.116 | 0.263 |
| rs2476601:114377568:A:G | 1 | 114377568 | A | G | 0.012 | 0.459 | 0.700 | 0.266 |
| rs187786174:2523811:G:A | 1 | 2523811 | G | A | 0.402 | 1.139 | 0.119 | 0.276 |
| rs2228145 | 1 | 154426970 | A | C | 0.910 | 1.272 | 0.223 | 0.279 |
| rs10985070 | 9 | 123636121 | C | A | 0.782 | 1.160 | 0.140 | 0.289 |
| rs9603616 | 13 | 40368069 | C | T | 0.789 | 1.156 | 0.142 | 0.307 |
| rs17668708:198640488:C:T | 1 | 198640488 | C | T | 0.946 | 0.782 | 0.241 | 0.307 |
| rs73194058:34764288:C:A | 21 | 34764288 | C | A | 0.984 | 0.627 | 0.465 | 0.315 |
| rs1571878 | 6 | 167540842 | C | T | 0.301 | 1.120 | 0.119 | 0.340 |
| rs9378815:426155:G:C | 6 | 426155 | G | C | 0.750 | 0.873 | 0.148 | 0.360 |
| rs7752903 | 6 | 138227364 | T | G | 0.948 | 0.816 | 0.230 | 0.377 |
| rs11605042:72411664:G:A | 11 | 72411664 | G | A | 0.541 | 1.108 | 0.116 | 0.380 |
| rs6732565 | 2 | 111607832 | A | G | 0.675 | 1.110 | 0.122 | 0.391 |
| rs4656942:160831048:G:A | 1 | 160831048 | G | A | 0.962 | 1.313 | 0.328 | 0.406 |
| rs508970:60906450:A:G | 11 | 60906450 | A | G | 0.136 | 0.868 | 0.171 | 0.407 |
| rs75409195:161644258:G:C | 1 | 161644258 | G | C | 0.997 | 48.613 | 4.681 | 0.407 |
| rs2236668:45650009:T:C | 21 | 45650009 | T | C | 0.834 | 0.881 | 0.156 | 0.415 |
| rs2234067:36355654:A:C | 6 | 36355654 | A | C | 0.015 | 0.610 | 0.613 | 0.420 |
| rs6715284:202154397:C:G | 2 | 202154397 | C | G | 0.571 | 0.914 | 0.116 | 0.435 |
| rs3218251:37545505:T:A | 22 | 37545505 | T | A | 0.642 | 0.915 | 0.119 | 0.454 |
| rs3783782:61940675:G:A | 14 | 61940675 | G | A | 0.996 | 0.527 | 0.897 | 0.476 |
| rs12413578 | 10 | 9049253 | C | T | 0.992 | 1.692 | 0.775 | 0.498 |
| rs4780401:11839326:G:T | 16 | 11839326 | G | T | 0.441 | 1.080 | 0.117 | 0.511 |
| rs9826828:136402060:G:A | 3 | 136402060 | G | A | 0.999 | 0.454 | 1.238 | 0.523 |
| 12:58108052:C:T | 12 | 58108052 | C | T | 0.603 | 0.930 | 0.114 | 0.526 |
| rs2105325:173349725:A:C | 1 | 173349725 | A | C | 0.112 | 0.895 | 0.188 | 0.554 |
| rs3778753 | 7 | 128580042 | A | G | 0.673 | 0.934 | 0.117 | 0.559 |
| rs28411352 | 1 | 38278579 | C | T | 0.798 | 0.926 | 0.138 | 0.576 |
| rs9372120 | 6 | 106667535 | T | G | 0.945 | 1.154 | 0.264 | 0.586 |
| rs12140275:38633879:A:T | 1 | 38633879 | A | T | 0.828 | 1.085 | 0.154 | 0.594 |
| rs138193887:107967350:A:G | 11 | 107967350 | A | G | 0.991 | 0.726 | 0.604 | 0.596 |
| rs678347:102463602:G:A | 8 | 102463602 | G | A | 0.350 | 1.064 | 0.119 | 0.599 |
| rs72717009:161405053:C:T | 1 | 161405053 | C | T | 0.836 | 1.087 | 0.161 | 0.605 |
| rs3806624 | 3 | 27764623 | A | G | 0.120 | 0.911 | 0.183 | 0.612 |
| rs73081554 | 3 | 58302935 | C | T | 0.993 | 1.475 | 0.776 | 0.616 |
| rs13385025:62461120:G:A | 2 | 62461120 | G | A | 0.869 | 0.924 | 0.168 | 0.636 |
| rs909685:39747671:T:A | 22 | 39747671 | T | A | 0.562 | 0.954 | 0.113 | 0.680 |
| rs72634030 | 17 | 5272580 | C | A | 0.988 | 1.303 | 0.644 | 0.681 |
| rs1950897 | 14 | 68760141 | C | T | 0.832 | 1.060 | 0.157 | 0.713 |
| rs7731626 | 5 | 55444683 | G | A | 0.835 | 1.057 | 0.153 | 0.716 |
| rs8083786:12881361:A:G | 18 | 12881361 | A | G | 0.443 | 1.043 | 0.116 | 0.718 |
| JHU_6.149834573 | 6 | 149834574 | C | T | 0.115 | 0.940 | 0.180 | 0.731 |
| rs17264332:138005515:A:G | 6 | 138005515 | A | G | 0.907 | 1.063 | 0.196 | 0.754 |
| rs8133843 | 21 | 36738242 | G | A | 0.289 | 0.961 | 0.129 | 0.755 |
| rs2469434:67544046:T:C | 18 | 67544046 | T | C | 0.454 | 1.036 | 0.113 | 0.757 |
| rs624988 | 1 | 117263790 | T | C | 0.543 | 0.966 | 0.115 | 0.764 |
| rs2233424 | 6 | 44233921 | C | T | 0.938 | 1.070 | 0.238 | 0.776 |
| rs45475795:123399491:A:G | 4 | 123399491 | A | G | 0.993 | 1.243 | 0.796 | 0.785 |
| rs9653442 | 2 | 100825367 | C | T | 0.726 | 0.966 | 0.127 | 0.788 |
| rs2301888 | 1 | 17672730 | G | A | 0.828 | 1.039 | 0.147 | 0.796 |
| rs13330176:86019087:T:A | 16 | 86019087 | T | A | 0.785 | 0.965 | 0.145 | 0.803 |
| rs227163 | 1 | 7961206 | C | T | 0.249 | 0.969 | 0.128 | 0.803 |
| 12:111833788:G:A | 12 | 111833788 | G | A | 0.034 | 0.919 | 0.344 | 0.806 |
| rs706778:6098949:C:T | 10 | 6098949 | C | T | 0.502 | 1.026 | 0.114 | 0.821 |
| rs2664035 | 4 | 48220839 | G | A | 0.564 | 1.024 | 0.112 | 0.834 |
| rs10175798 | 2 | 30449594 | G | A | 0.736 | 0.973 | 0.129 | 0.835 |
| rs1893592 | 21 | 43855067 | A | C | 0.947 | 0.954 | 0.253 | 0.852 |
| rs34695944 | 2 | 61124850 | T | C | 0.934 | 1.034 | 0.235 | 0.889 |
| rs1516971 | 8 | 129542100 | T | C | 0.626 | 0.986 | 0.117 | 0.904 |
| rs2317230:157674997:G:T | 1 | 157674997 | G | T | 0.080 | 0.982 | 0.230 | 0.937 |
| rs11574914 | 9 | 34710338 | G | A | 0.931 | 0.986 | 0.219 | 0.949 |
| rs59716545:38031857:T:G | 17 | 38031857 | T | G | 0.880 | 0.989 | 0.176 | 0.949 |
| rs968567:61595564:C:T | 11 | 61595564 | C | T | 0.984 | 1.023 | 0.458 | 0.960 |
| rs726288:81706973:C:T | 10 | 81706973 | C | T | 0.991 | 1.033 | 0.658 | 0.960 |
| rs4272 | 7 | 92236829 | A | G | 0.816 | 1.001 | 0.144 | 0.993 |
| rs13142500:10727357:T:C | 4 | 10727357 | T | C | 0.667 | 1.001 | 0.125 | 0.996 |
| rs147622113:10771941:C:T | 19 | 10771941 | C | T | 1.000 | NA | NA | NA |

Trans-ethnic meta-analysis by Okada *et al.*

**Supplementary Table S3. Associations with Smoking and Alcohol Consumption in ACPA-Positive African Ancestry RA Cases**

|  | **GENRA** | **SLESS** |  |  |
| --- | --- | --- | --- | --- |
|  | **Characteristic** | **Characteristic** | **OR (95% CI)** | ***P*-value** |
| Age, mean (SD) | 57.5 (15.4) | 58.6 (12.6) | 1.00 (0.97-1.01) | 0.28 |
| Female, n (%) | 110 (83.3) | 220 (83.3) | 1.21 (062-2.37) | 0.57 |
| Caribbean ethnicity, n (%) | 79 (59.8) | 166 (62.9) | 0.86 (0.54-1.36) | 0.52 |
| Ever-Smoker, n (%) | 41 (31.1) | 57 (21.6) | 2.13 (1.24-3.66) | 6.0x10^-3^ |
| Alcohol Drinker, n (%) | 21 (15.9) | 89 (33.7) | 0.30 (0.17-0.53) | 3.9x10^-5^ |

**Supplementary Table S4. Univariate Estimates of the Influence of Smoking and Alcohol Consumption on RA Risk in African Ancestry Cases/Controls**

|  | **GENRA** | **SLESS** | |  |
| --- | --- | --- | --- | --- |
|  | **Characteristic** | **Characteristic** | **OR (95% CI)** | ***P*-value** |
| Ever-Smoker, n (%) | 54 (31.8) | 59 (20.3) | 1.83 (1.20-2.78) | 0.0046 |
| Alcohol Drinker, n (%) | 28 (16.5) | 108 (31.8) | 0.42 (0.27-0.67) | 0.00030 |
